# Characterization of New Flavored Oils Obtained Through the Co-Milling of Olives and Vegetable Food Products

**DOI:** 10.3390/foods14040687

**Published:** 2025-02-17

**Authors:** Celeste Lazzarini, Matilde Tura, Mara Mandrioli, Marco Setti, Noureddine Mokhtari, Abdelaziz Ait Elkassia, Sara Barbieri, Enrico Valli, Alessandra Bendini, Tullia Gallina Toschi

**Affiliations:** 1 Department of Agricultural and Food Sciences (DISTAL), Alma Mater Studiorum—Università di Bologna, Viale Fanin, 40, 40127 Bologna and Piazza Goidanich, 60, 47521 Cesena, Italy; celeste.lazzarini3@unibo.it (C.L.); matilde.tura2@unibo.it (M.T.); mara.mandrioli@unibo.it (M.M.); marco.setti@unibo.it (M.S.); sara.barbieri@unibo.it (S.B.); alessandra.bendini@unibo.it (A.B.); tullia.gallinatoschi@unibo.it (T.G.T.); 2 Ecole Nationale d’Agriculture de Meknès, Meknès 50001, Morocco; nmokhtari@enameknes.ac.ma (N.M.); aziz_iaa@hotmail.com (A.A.E.)

**Keywords:** flavored olive oil, sustainability, co-milling, hemp, orange, black pepper

## Abstract

Consumers are increasingly attracted to innovative, gourmand, and sustainable food products. This has led to a growing interest in flavored olive oils through co-milling processing. This study explores the production and characterization of flavored olive oils obtained by co-milling olives with orange pomace, black pepper, and hemp seeds, aiming to enhance their sensory and compositional properties while promoting sustainability through the valorization of agri-food by-products. The flavored olive oils and their control samples were analyzed for free acidity, tocopherols, phenolic compounds, volatiles, and sensory profiles. The flavored oils exhibited an acceptable hydrolytic state and peculiar sensory notes, depending on the ingredients used, as well as enhanced compositional qualities. This research highlights the potential of using oranges and hemp by-products in flavored oil production, offering an innovative approach to reducing food waste, with the possibility of future industrial applications.

## 1. Introduction

Extra virgin olive oil (EVOO) is the main source of fats for the Mediterranean diet, making it possible to consider it a healthy nutritional model [1]. EVOO is commonly recognized for its nutritional qualities and oxidative stability, thanks to its composition and richness in bioactive compounds such as phenols [2]. In addition, its health benefits are related to the high content of monounsaturated fatty acids, in particular oleic acid, and to the ratio of saturated and polyunsaturated fatty acids [3]. The importance of phenolic compounds in olive oil is underscored by the health claim established by the European Commission Regulation 432/2012, and its subsequent amendments, which specifically applies to virgin olive oils. This regulation states that “olive oil polyphenols contribute to the protection of blood lipids from oxidative stress” [4], highlighting their significant role in promoting cardiovascular health. Polyphenols are not only valued for their health benefits but are also key contributors to the sensory profile of high-quality olive oils. These molecules are responsible for the characteristic pungency and bitterness that are highly prized in EVOO. In addition to these sensory attributes, the most important positive descriptor is fruity.

Other secondary positive sensory notes include a range of aromas and flavors that reflect both the cultivar of the olive and the region of production. These descriptors commonly include hints of tomato, as well as fresh-cut grass, green herbs, almond, and artichoke, as also reported in an IOC document on the methods to be used for the organoleptic assessment of EVOO for the purposes of DO status [5,6].

Aromatic plants, spices, herbs, fruits, and vegetables have been used, since ancient times, in food flavoring, pharmaceutical, and cosmetics fields due to their biological activity, including antioxidant properties, and sensory characteristics [3].

The use of flavored olive oils has gained importance in the last decades, especially in non-Mediterranean countries, produced by adding different aromas coming from various plant species, herbs, and essential oils to mask and mitigate the strong pungent and bitter attributes, which are not always appreciated by consumers [7,8].

The flavoring of olive oils, with the use of different techniques, affects both the sensory and physicochemical characteristics of the oils, by adding new aromas and enhancing oxidative stability, improving the oil shelf-life [9,10]. Aromatization usually involves ingredients such as basil, chili pepper, essential oils for *Lamiaceae*, other vegetables such as garlic and onions, and herbs like oregano, rosemary, and sage, as well as fruits (in particular banana, citrus, and apple) [11]. Even if flavored olive oils are now gaining importance, this is an ancient practice, especially in the Mediterranean region, which can follow three techniques: the contact method, co-extraction, or co-milling with the incorporation of essential oils [10]. In the co-extraction technique, the one used for this work, the flavoring agent is directly added to the olives in the milling phase, while during the malaxation stage, the large contact surface allows for the migration of compounds from the flavoring agent to the olive pomace [12]. The most common flavoring agents are citruses, such as lemons and oranges, and tangerines. In the case of citrus, the part richest in essential oils is the skin, which can be directly added to the olives [13].

According to Khemakhem et al. (2015) [3], the addition of citrus zest can increase the polyphenol and carotenoid contents, improving the antioxidant activity and the aroma.

Indeed, as described, the addition of flavorings to olive oil can be advantageous for both the sensory and the compositional aspects by enhancing desirable flavors and improving oxidative stability and shelf-life [14]. As demonstrated by Díaz-Montaña and colleagues (2022), the addition of basil (*Ocimum basilicum* L.) and rosemary (*Rosmarinus officinalis* L.) to virgin olive oils slowed down the oxidation process with modification of the phenolic fraction [15]. Similarly, not only herbs but also fruits have shown such potential; also, bergamot added to olive oil promoted inhibitory activity against key enzymes linked to obesity, as well as scavenging activity, while also being appreciated by consumers [16].

The use of by-products, coming from fruit juice production, can play an important role in the valorization of food products, while also transferring important bioactive compounds to olive oil. This promotes a circular economy while producing a valuable product that is now more and more important in the market.

In fact, orange juice is the most popular fruit beverage around the world [17], and Brazil and the USA (especially Florida) are the largest producers, with 1 and 0.5 metric tons, respectively, since 2011 (USDA/FAS 2015) [18]. Nearly 50% of the fruit is considered waste, accounting for 14 million tons globally [19], which is commonly used as a supplement for animal feed or for the preparation of pellets [20].

*Cannabis sativa* L. is a versatile plant, providing material for foods, textiles, fibers, and food supplements, and the pharmaceutical field [21]. Hemp seeds have been considered by-products, but quite recently, thanks to their nutritional properties, have gained growing importance in the food system [22]. Hemp seed contains approximately 25% to 30% oil, 25% to 30% protein, 30% to 40% fiber, and 6% to 7% moisture; moreover, the balance between *ω*-6/*ω*-3 fatty acids is considered optimal from a nutritional point of view [23]. In addition, hemp seed oil is an important source of other beneficial compounds that have a positive effect on the human cardiovascular system, which cannot be produced by the human body, namely linoleic acid and α-linolenic acid [24].

In the present study, the sensory and compositional characteristics of different oils were assessed, namely three olive oils, with control samples, and co-milled olive oils with oranges, orange by-product, and orange by-product plus black pepper and hemp seeds, respectively.

This preliminary study not only lays the groundwork for future industrial applications but also presents an innovative opportunity to valorize food by-products, thus contributing to more sustainable and circular resource management. The herein presented investigation is focused on obtaining and characterizing new high-added-value flavored oils that could be produced on a large scale and possibly sold in the market.

## 2. Materials and Methods

### 2.1. Oil Production

**Figure 1:**
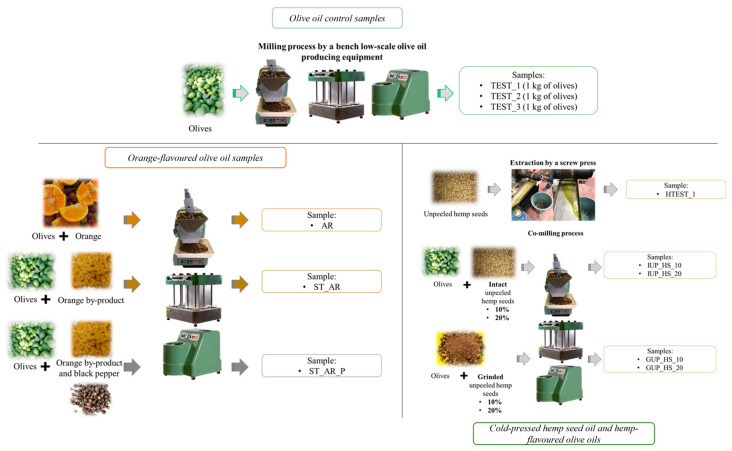
Description of the samples produced and analyzed in this research work.

By using the lab scale mill Abencor^®^ (MC2 Ingeniería y Sistemas S.L, Sevilla, Spain), 5 different oils were produced: 3 control samples, with olives only, collected from the campus of Cesena and from Brisighella fields, processed immediately after collection (namely TEST_1 and TEST_3, produced with olives collected from the campus of Cesena, and TEST_2, produced with the olives collected from fields in Brisighella), while a control sample of cold-pressed hemp seed oil (HTEST_1) was produced by using a screw press (KK20, Kern Kraft, Reut, Germany). Moreover, flavored oils with different matrices, in particular, entire oranges (sample AR, 1 kg of olives + 150 g of entire oranges); an orange by-product (ST_AR, 1 kg of olives + orange by-product deriving from the lab-scale juicing of 350 g of oranges through a manual screw press) with TEST_1 as the control sample; orange by-product and black pepper (ST_AR_P, 1 kg of olives + orange by-product deriving from the juicing of 350 g of oranges + 10 g of black pepper) with TEST_2 as the control sample; intact unpeeled hemp seeds at 10 and 20% (samples IUP_HS_10 and IUP_HS_20, respectively) and ground unpeeled hemp seeds at 10 and 20% (samples GUP_HS_10 and GUP_HS_20, respectively) with TEST_3 as the control sample. Hemp seeds, of the variety Futura 75, were collected from a local company and stored at 12 °C before processing. Such oils were produced by co-milling, thus crushing olives directly with the listed matrices (Table 1 and Figure 1).

### 2.2. Chemicals and Reagents

All chemicals used were of analytical grade. Diethyl ether CAS 60-29-7 (ACS reagent, purity ≥ 99.8%), ethanol CAS 64-17-5 (ACS reagent, purity ≥ 96%), 4-methyl-2-pentanol CAS 108-11-2 (purity > 95%), methanol CAS 67-56-1 (purity ≥ 99.8%), Folin–Ciocalteu’s reagent CAS 12111-13-6, sodium carbonate CAS 144-55-8 (purity ≥ 99.8%), gallic acid CAS 149-91-7, γ-tocopherol CAS 54-28-4 (analytical standard), α-tocopherol CAS 119-13-1 (analytical standard), and δ-tocopherol CAS 119-13-1 (analytical standard) were purchased from Sigma-Aldrich (St. Louis, MI, USA).

Sodium hydroxide 0.1 N CAS 1310-73-2 and phenolphthalein 1% in ethanol CAS 77-09-8 were purchased from Carlo Erba Reagents S.r.l. (Milan, Italy).

### 2.3. Free Acidity

To determine the free acidity, the method reported by the European Regulation 2104/22 [25] was followed for virgin olive oil control samples as well as for the flavored samples, even if they cannot be commercialized as extra virgin, virgin, or lampante olive oil, and for the hemp seed oil control sample.

An aliquot (g) of the oil sample was dissolved in 100 mL of a solution of diethyl ether and ethanol (1:1 *v*/*v*, previously neutralized), and free fatty acids were neutralized using sodium hydroxide (0.1 mol/L) as the titering solution and 1% phenolphthalein in ethanol as the indicator solution. Two analytical replicates were performed for each sample.

### 2.4. Tocopherol Content

The determination was performed by liquid chromatography coupled with a diode-array detector (HPLC-DAD). Initially, 0.5 g was solubilized in isopropanol, and 20 µL was injected into an RP-HPLC system equipped with a quaternary pump model HP 1260 and diode-array detector; the software for data processing was Chemstation for LC3D (Agilent Technologies, Santa Clara, CA, USA). The instrument was equipped with a Cosmosil π NAP 150 mm × 4.6 mm column, 5 µm (Nacalai-Tesque, Kyoto, Japan). The mobile phase and the elution gradient were the same as those reported by UNI/TS 11825:2021. The diode-array detector was set up at 292 nm. Quantification was carried out using calibration curves of α- and γ-tocopherols (CAS numbers 10191–41–0 and 54–28–4, respectively; Sigma-Aldrich, St Louis, MO, USA), which were constructed with the external standard method, injecting solutions of known concentration in the range of 0.5–50 mg/L. The equation of the calibration curve was y = 8.2451x − 5.2057 (r^2^ = 0.999) for α-tocopherol. γ-tocopherol and δ-tocopherol were identified using the related standards, and they were quantified using the calibration curve of α-tocopherol. Three analytical replicates were performed for each sample.

### 2.5. Content of Molecules with Reducing Activity

To determine the content of molecules with reducing activity, a colorimetric approach was used. This assay is based on the redox reaction of the Folin–Ciocalteu (Sigma Aldrich, Darmstadt, Germany) reagent with hydroxyl groups or with the reducing activity molecules present in olive oil hydro-alcoholic (methanol:water 80:20 *v*:*v*%) (Sigma Aldrich, purity ≥ 99.8%) extracts. The procedure implies the spectrophotometric analysis of the diluted extracts after alkalinization with sodium carbonate (15%) (Sigma-Aldrich, purity ≥ 99.8%) and redox reaction with Folin–Ciocalteu reagent. The calibration curve for the quantification was prepared with gallic acid (Sigma Aldrich). Three analytical replicates were performed for each sample.

### 2.6. Volatile Compound Analysis by SPME-GC-MS

This determination was performed by solid-phase microextraction coupled with gas chromatography–mass spectrometry (SPME/GC–MS) (QP2010 Ultra, Shimadzu, Kyoto, Japan) with the autosampler (AOC-5000 plus, Shimadzu). The method described by Aparicio-Ruiz et al. (2022) [26] was followed with respect to the sample preparation and analysis conditions, and peak identification was tentatively based on comparing mass spectrum data with spectra present in the National Institute of Standards and Technology library 2008 version (NIST^®^08) and taking into account Linear Retention Indices (Kovats indexes). An aliquot of 1.9 g of oil added with 0.1 g of internal standard (4-methyl-2-pentanone dissolved in refined olive oil at 50 mg/kg) was placed in a 20 mL vial sealed with a polytetrafluoroethylene (PTFE) septum and maintained at 40 °C under agitation for 10 min to allow the volatiles to equilibrate within the headspace. Subsequently, the SPME fiber (length 1 cm, 50/30 μm f.t. endowed with a stationary phase divinylbenzene/carboxen/polydimethylsiloxane) was exposed to the headspace at 40 °C for 40 min. Once this process was complete, the fiber was introduced into the injector port of the GC. The volatiles captured by the fiber were thermally released in the heated injection port of a GC at 250 °C for 5 min in splitless mode (purge valve off) and subsequently injected into a capillary column connected to the gas chromatograph equipped with the mass spectrometry detector. The capillary column featured a polar phase based on polyethylene glycol (PEG) with a length of 60 m, an internal diameter of 0.25 mm, and a coating thickness of 0.50 μm. The MS transfer line temperature was set at 260 °C and helium was used as carrier gas (flow: 1.5 mL/min). The oven temperature was held at 40 °C for 10 min and then programmed to increase by 3 °C/min to a final temperature of 200 °C. A cleaning step was added at the end of the programmed oven temperature (20 °C/min to 250 °C for 5 min). For the quantification, the concentration of the internal standard was used together with the chromatographic area of the analyte. Three analytical replicates were performed for each sample.

### 2.7. Descriptive Sensory Analysis

The sensory analysis was carried out by the University of Bologna professional committee which is recognized by the Italian Ministry of Agriculture, Food Sovereignty and Forestry for the sensory analysis of VOOs. For olive oil control samples (TEST_1 and TEST_2) and for the co-milled ones with orange and orange and pepper, the sensory evaluation was performed according to the rules established by IOC/T.20/Doc. No 15 (Rev 11.2024) [5]. Panelists were also asked to assess secondary positive attributes (IOC/T.20/Doc. no 22) [27].

For cold-pressed hemp seed oil, olive oil control sample TEST_3, and co-milled olive and hemp seed oil samples, sensory evaluation was made following a rapid descriptive method, i.e., flash profile. In particular, three sensory sessions were performed. During the first one, assessors were asked to generate the vocabulary, including non-hedonistic terms that, in their opinion, most described the samples and that allowed for the classification of the samples according to the intensity of each selected attribute. Then, an open discussion among the judges was held; during this phase, each assessor could keep their own vocabulary or add, delete, or rename attributes. Finally, the judges were asked to rank the samples according to the perceived intensity of each attribute [28]. Regarding the sensory evaluation of the control olive oil sample TEST_3, cold-pressed hemp seed oil, and co-milled oils of olives and hemp, only the two control samples (TEST_3 and HTEST_1) and the two samples produced with ground olives and seeds (GUP_HS_10 and GUP_HS_20) were subjected to sensory analysis.

### 2.8. Data Processing and Statistical Analysis

Data processing and calculation were carried out with Microsoft^®^ spreadsheet program 2016 (Microsoft Corp., Redmond, WA, USA). Analysis of variance (analysis of variance (one-way ANOVA, Tukey’s HSD, *p* < 0.05), GPA, and MFA were performed with XLSTAT (Addinsoft Corp., Paris, France).

## 3. Results

This section may be divided by subheadings. It should provide a concise and precise description of the experimental results, their interpretation, as well as the experimental conclusions that can be drawn.

### 3.1. Assessment of the Hydrolytic State

According to the European Regulation (EU) 2019/1604 [29], olive oils can be classified as extra virgin when the free acidity is 0 ≤ 0.8 g per 100 g of oil (0.8%) expressed in oleic acid. Whereas, according to the Codex Alimentarius, the free acidity of vegetable oils should be under 4 mg KOH/g of oil (CODEX STAN 19-1981) [30].

The virgin olive oils produced for this investigation show a lower level of percentage of oleic acid with respect to the co-milled ones, as reported in Table 2, in such a way that the two control samples (Test 1 and Test 2) fall into the category of extra virgin olive oils. This indicates the low hydrolysis of triglycerides. For the co-milled samples that cannot be qualified as “extra virgin” due to the addition of ingredients to olives, free acidity values provide valuable information about the raw material hydrolytic conditions and the related overall stability. Indeed, a sufficiently low free acidity suggests that the co-milled oils have maintained an acceptable quality of the hydrolytic level. The same can be stated for the olive oils co-milled with hemp seeds, for which the hydrolytic degradation is below 4.0 mg KOH/g of oil, as suggested by the Codex Alimentarius [31].

### 3.2. Content of Molecules with Reducing Activity

**Figure 2:**
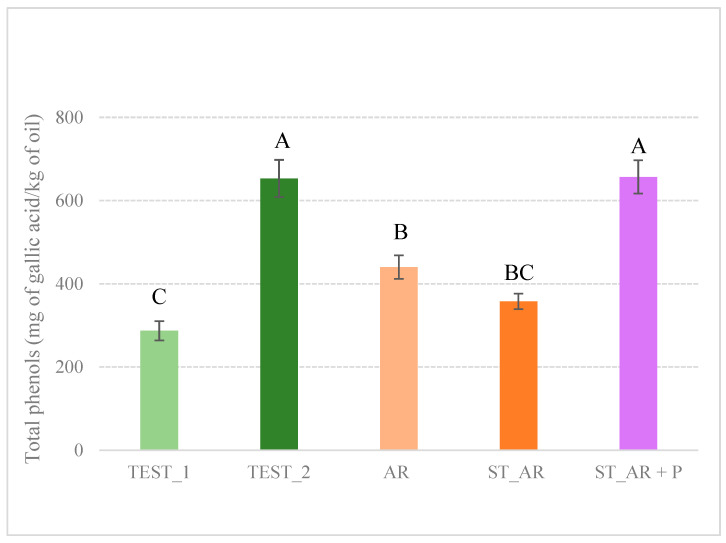
Total content of reducing activity molecules expressed as mg of gallic acid per kg of oil. Letters of significance are related to the analysis of variance (ANOVA, Tukey’s HSD (*p* ≤ 0.05). TEST_1, TEST_2 = olive oil control samples; AR = co-milled olive oil produced by milling olives and entire oranges; ST_AR = co-milled olive oil produced by milling olives and orange pomace; ST_AR_P = co-milled olive oil produced by milling olives with orange pomace and black pepper.

The contents of molecules with reducing activity are shown in Figure 2. The differences between the two control samples (TEST_1 282.26 mg/kg of oil and TEST_2 653.09 mg/kg of oil) can be related to a different lot of processed olives.

According to the classification described in Montedoro et al. (1992) [32] for virgin olive oils, the oils can all be categorized as having medium and high total phenol content; in particular, TEST_1 (287.26 mg/kg), AR (440.29 mg/kg), and ST_AR (357.67 mg/kg) can be considered as having medium phenol content (200–500 mg/kg), while TEST_2 and ST_AR_P. (with 653.09 mg/kg and 656.92 mg/kg, respectively) can be classified as having high total phenol content (500–1000 mg/kg) [32].

These results align with the oil preparation methods, as TEST_2 exhibits a comparable level of phenolic compounds to ST_AR_P, prepared using such a control sample as a base. However, it seems that phenolic compounds are not effectively transferred from the vegetable matrices to the oil, particularly orange pomace and pepper, during the production process, and this has also occurred for ST_AR and AR. Such compounds, particularly abundant in citrus, may show different affinities for different olive varieties and maturity, making them more difficult to transfer. Additionally, the lipidic composition in different olive batches may be less effective in dissolving and retaining these compounds [33,34]. The results are consistent with the findings of Chahdoura and colleagues (2023), where orange-flavored olive oil (produced in a similar manner) exhibited a comparable increase in total phenolic compounds from the control sample to the flavored one [35].

### 3.3. Tocopherol Profile

Several minor compounds, including tocopherols, chlorophylls, carotenes, and cannabinoids, are typically present in cold-pressed hemp seed oil.

Cannabinoids were previously evaluated in a study where their presence in seeds was detected only in trace amounts, below the permitted limit. Therefore, such monitoring should be conducted prior to the use of hemp seeds for co-milling.

Tocopherols are known to prevent PUFA-rich oils, such as cold-pressed hemp seed oil, from oxidation, preserving the lipid matrix [36]. The main tocopherol of hemp seed oil is typically γ-tocopherol [37], while the main one in olive oil is α-tocopherol [38]. Given the potential synergistic effects between compounds from different plant sources, the analysis of tocopherols was focused only on the co-milled samples to assess the impact of combining hemp seeds and olives on the overall profile.

The results are in line with the existing literature: The main tocopherol found in sample TEST_3 (olive oil control sample) was α-tocopherol, while the main one in cold-pressed hemp seed oil (HTEST_1) was γ-tocopherol (Table 3).

The samples obtained from the co-milling of olives and hemp seeds showed a lower content of γ-tocopherol when the sample was obtained from a lower percentage of seeds (10% *w*/*w*) and when the seeds were whole. In fact, the sample with the lowest γ-tocopherol content was IUP_HS_10, while the one with the highest content was sample GUP_HS_20 (Table 2). On the other hand, due to a high percentage of olives (*w*/*w*), sample IUP_HS_10 also showed a higher content of α-tocopherol in comparison with the other co-milled samples (Table 3).

### 3.4. Volatile Compound Analysis

Concerning virgin olive oils (control samples) and flavored oils, the concentrations of the main volatile compounds, which were tentatively identified with the use of LRIs compared to the values reported in the NIST library and in the literature [39,40,41,42,43], are reported in Table 4 and Table 5.

Different concentrations of volatile compounds in the two extra virgin olive oil samples can be related to agronomic aspects, among them different cultivars, geographic origin, and the ripening state of the fruit [44]. Indeed, TEST_1 and TEST_2 were produced by crushing two batches of different olives coming from different fields (TEST_1 Cesena, TEST_2 Brisighella). Both oils present volatile compounds related to positive attributes such as fruity and green ((*E*)-2-hexenal, *(Z)*-3-hexen-1-ol, (*E*)-2-hexen-1-ol, 1-penten-3-one, 3-ethyl-1,5-octadiene) [45,46].

In the sample obtained by co-milling with entire oranges (AR), several volatile compounds present in the oranges were transferred to the flavored oil itself, with possible consequences for the sensory profile. Indeed, such samples were clearly recognized by the sensory panel as orange-flavored. Among the detected volatile compounds, α-pinene, 1-penten-3-one, sabinene, limonene, β-myrcene, β-phellandrene, and octanal are the most abundant and peculiar in orange juice [47,48]. In addition, limonene, β-myrcene, and β-phellandrene are highly volatile hydrophobic terpenes soluble in oils that are able to be trapped in oil droplets during its production by co-milling [35,49]. The same compounds were detected in olive oil flavored by co-milling with orange pomace (ST_AR), even if in different concentrations.

Regarding sample ST_AR_P (olives co-milled with orange by-product and black pepper), compounds related to the orange fruit were detected, similar to the other orange-flavored samples, as well as terpenes from black pepper; in particular, β-pinene, α-pinene, sabinene, β-myrcene, γ-terpinene, β-(*Z*)-ocimene, limonene, linalool, and 3-thujene. Such compounds can contribute to pleasant aromatic notes, namely balsamic, citrus, and peppermint scents, usually also present in *Cannabis sativa* L. and derived products that have, in addition, antioxidant and inflammatory properties [50,51,52].

Terpenes are compounds typically found in hemp, produced in glandular trichomes, and exhibit an entourage effect (synergistic action) with cannabinoids [53].

The profile of volatile compounds (Table 5) highlights the presence of peculiar terpenic compounds in the hemp seed oil samples and in the co-milled ones, such as α-pinene, β-pinene, 3-carene, limonene, β-(*Z*)-ocimene, and caryophyllene, which have already been previously identified by other authors [37,54,55] in hemp seed oils. On the other hand, sample TEST_3 (virgin olive oil) was characterized by volatile compounds generally found in olive oils, such as 2-methyl-butanal, 3-methyl-butanal, 3-pentanone, hexanal, 3-methyl-butanol, (*E*)-2-hexenal, and (*Z*)-3-hexenol [56,57,58]. The co-milled samples with the highest concentration of terpenes were those obtained from co-milling olives and ground hemp seeds at 20% (*w*/*w*). Additionally, the presence of (*E*)-2-hexenal in hemp seeds and their co-milling with olives led to a higher concentration of this compound in the flavored samples compared to the control sample [59].

### 3.5. Sensory Analysis

According to the Reg. (EU) 2022/2104 and Reg. (EU) 1169/2011 [21,60], flavored oils do not fall within the commercial categories of virgin olive oils. Therefore, for the flavored oils presented here, the sensory evaluation by the panel can provide indications regarding their quality status, highlighting certain sensory characteristics (e.g., secondary positive attributes).

According to the sensory analysis, the produced oils do not present any defects, and the intensities of positive attributes are reported in Table 6.

As described in the previous paragraph, the differences between the two control samples can be related to the different batches of olives used. Median values of pungency and bitterness are coherent with the results obtained from the analysis of molecules with reducing activity: TEST_2 shows a higher concentration of molecules with reducing activity and the highest bitterness and pungency intensities. The judges perceived sensory notes of citrus for each orange-flavored oil; in addition, the flavoring agents may have masked the fruity attribute related to fresh olives.

**Figure 3:**
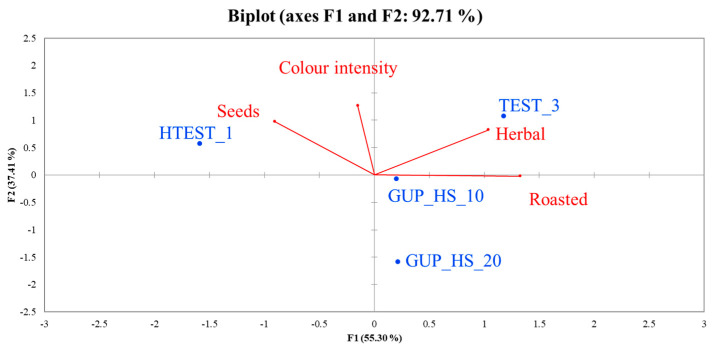
Generalized Procrustes Analysis (GPA) biplot of sensory data (flash profile method) of co-milled olive oils with hemp seeds at different ratios and their control samples. TEST_3 = olive oil control sample; HTEST_1 = cold-pressed hemp seed oil control sample; IUP_HS_10 = co-milled olive oil produced by milling olives with 10% intact unpeeled hemp seeds; IUP_HS_20 = co-milled olive oil produced by milling olives with 20% intact unpeeled hemp seeds; GUP_HS_10 = co-milled olive oil produced by milling olives with 10% ground unpeeled hemp seeds; GUP_HS_20 = co-milled olive oil produced by milling olives with 20% ground unpeeled hemp seeds.

Regarding the oils co-milled with different ratios of hemp seeds, only those obtained with ground hemp seed and the two control samples (virgin olive oil and cold-pressed hemp seed oil) were subjected to sensory analysis using the flash profile method. The results of the sensory evaluation were processed using Generalized Procrustes Analysis (GPA) (Figure 3). The distribution of the samples on the plane based on sensory attributes shows that the sample HTEST_1 is positioned near the attribute seeds; TEST_3 is characterized by the attribute herbaceous; GUP_HS_10 and GUP_HS_20 are more like each other and characterized by a low intensity of the attribute color intensity (from yellow to green).

### 3.6. Joint Elaboration of Volatile Compounds and Sensory Data

**Figure 4:**
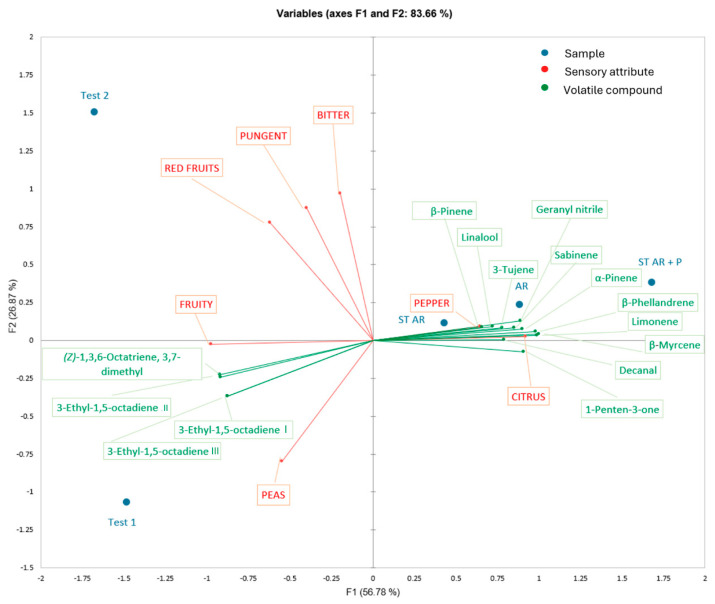
Multiple Factor Analysis (MFA) biplot obtained by volatile compounds (in green) and sensory data (median intensity of sensory attributes, in red) of flavored olive oils with orange, orange by-product, orange by-product and black pepper, and their control samples. TEST_1, TEST_2 = olive oil control samples; AR = co-milled olive oil produced by milling olives and entire oranges; ST_AR = co-milled olive oil produced by milling olives and orange pomace; ST_AR_P = co-milled olive oil produced by milling olives with orange pomace and black pepper.

The biplot obtained by the MFA analysis (Figure 4) of selected volatile compounds concentrations and sensory data shows a clear clusterization among the oils: TEST_1 is mainly characterized by isomers of 3-ethyl-1,5-octadiene and 3,7-dymethyl-1,3,6-octatriene; both AR and ST_AR are mainly characterized by the presence of orange-derived volatile compounds (limonene, β-myrcene, geranyl nitrile, and decanal). The flavored olive oil obtained with orange by-product and black pepper are characterized by the presence of β-pinene, sabinene, 3-thujene, and volatile compounds characterizing the orange-flavored samples (Figure 4).

## 4. Conclusions

The characterization of co-milled olive oils with different flavoring matrices, namely orange, orange pomace, black pepper, and hemp seeds, highlighted interesting aspects of their composition and sensory profile. The incorporation of orange by-products into olive oil increased the phenolic content, contributing to the sensory profile. The samples co-milled with orange and orange by-products displayed medium to high total phenol content, suggesting a significant transfer of bioactive compounds from the flavoring agents to the olive oil. The tocopherol profiles differed between the control sample and hemp seed oil samples. The co-milled samples with hemp seeds showed variable levels of α- and γ-tocopherols, influenced by the percentage and form of the hemp seeds used. The highest γ-tocopherol content was found in samples with ground hemp seeds at a 20% ratio. In addition, such products showed various terpenes typical of hemp, with bioactive activity, such as pinene and limonene. Co-milling, a widely used technique, facilitated the migration from the flavoring matrices to the oil of different compounds with well-known beneficial properties for both human health and sensory perception by producing new so-called gourmet oils, meeting consumers’ demand. This research introduces an innovative approach to the existing co-milling technique by incorporating by-products as a flavoring matrix, thereby significantly enhancing the sustainability and circularity of this process. It is essential to characterize these new products and evaluate their qualitative and compositional attributes, which otherwise would remain underexplored. The industrial interest around this sustainable technique supports the production of gourmet oils, simultaneously lowering production costs and improving overall sustainability.

## Figures and Tables

**Table 1:** Summary of the samples and their description.

Sample Code	Description
TEST_1	Olive oil control sample 1 produced by milling olives collected from campus of Cesena
TEST_2	Olive oil control sample 2 produced by milling olives collected from fields of Brisighella
AR	Co-milled olive oil produced by milling olives collected from campus of Cesena (TEST_1) with entire oranges
ST_AR	Co-milled olive oil produced by milling olives collected from campus of Cesena (TEST_1) with orange pomace
ST_AR_P	Co-milled olive oil produced by milling olives collected from fields of Brisighella (TEST_2) with orange pomace and black pepper
TEST_3	Olive oil control sample 3 produced by milling olives collected from campus of Cesena
HTEST_1	Control sample of cold pressed hemp seed oil produced from Futura 75 hemp seeds collected in Italy
IUP_HS_10	Co-milled olive oil produced by milling olives collected from campus of Cesena (TEST_3) with intact hemp seeds (10%)
IUP_HS_20	Co-milled olive oil produced by milling olives collected from campus of Cesena (TEST_3) with intact hemp seeds (20%)
GUP_HS_10	Co-milled olive oil produced by milling olives collected from campus of Cesena (TEST_3) with ground hemp seeds (10%)
GUP_HS_20	Co-milled olive oil produced by milling olives collected from campus of Cesena (TEST_3) with ground hemp seeds (20%)

**Table 2:** Free acidity (% oleic acid) of control samples and flavored olive oils. Letters of significance are related to the analysis of variance (ANOVA, Tukey’s HSD (*p* ≤ 0.05). Lowercase letters refer to the ANOVA computed for hemp seeds, co-milled olive oils, and their control samples, while capital letters refer to the ANOVA computed for the set of samples involving the use of orange and black pepper and their control samples. TEST_1, TEST_2, TEST_3 = olive oil control samples; AR = co-milled olive oil produced by milling olives and entire oranges; ST_AR = co-milled olive oil produced by milling olives and orange pomace; ST_AR_P = co-milled olive oil produced by milling olives with orange pomace and black pepper; HTEST_1 = cold-pressed hemp seed oil control sample; IUP_HS_10 = co-milled olive oil produced by milling olives with 10% intact unpeeled hemp seeds; IUP_HS_20 = co-milled olive oil produced by milling olives with 20% intact unpeeled hemp seeds; GUP_HS_10 = co-milled olive oil produced by milling olives with 10% ground unpeeled hemp seeds; GUP_HS_20 = co-milled olive oil produced by milling olives with 20% ground unpeeled hemp seeds.

Sample	Free Acidity (% Oleic Acid)	Free Acidity (mg KOH/g of Oil)
TEST_1	0.25 ± 0.00 ^BC^	-
TEST_2	0.23 ± 0.00 ^C^	-
AR	0.26 ± 0.01 ^B^	
ST_AR	0.39 ± 0.01 ^A^	
ST_AR_P	0.38 ± 0.00 ^A^	
TEST_3	0.35 ± 0.01 ^d^	0.69 ± 0.02 ^d^
HTEST_1	0.94 ± 0.07 ^b^	1.86 ± 0.14 ^b^
IUP_HS_10	1.24 ± 0.00 ^a^	2.47 ± 0.00 ^a^
IUP_HS_20	0.65 ± 0.02 ^c^	1.29 ± 0.04 ^c^
GUP_HS_10	0.71 ± 0.01 ^c^	1.42 ± 0.02 ^c^
GUP_HS_20	1.03 ± 0.00 ^b^	2.05 ± 0.00 ^b^

**Table 3:** Tocopherol (δ, γ, α) contents (mg α -tocopherol/kg oil) in olive oils co-milled with hemp seeds. Significance letters refer to the ANOVA, Tukey’s HSD (α = 0.05). TEST_3 = olive oil control sample; HTEST_1 = cold-pressed hemp seed oil control sample; IUP_HS_10 = co-milled olive oil produced by milling olives with 10% intact unpeeled hemp seeds; IUP_HS_20 = co-milled olive oil produced by milling olives with 20% intact unpeeled hemp seeds; GUP_HS_10 = co-milled olive oil produced by milling olives with 10% ground unpeeled hemp seeds; GUP_HS_20 = co-milled olive oil produced by milling olives with 20% ground unpeeled hemp seeds.

Sample	δ-Tocopherol mg/kg	γ-Tocopherol mg/kg	α-Tocopherol mg/kg
TEST_3		21.21 ± 1.17 ^e^	295.66 ± 0.86 ^a^
HTEST_1	12.58 ± 0.06 ^a^	817.49 ± 1.18 ^a^	54.99 ± 1.56 ^d^
IUP_HS_10		100.24 ± 0.97 ^d^	234.93 ± 2.17 ^b^
IUP_HS_20		256.65 ± 4.67 ^b^	213.25 ± 5.19 ^c^
GUP_HS_10		142.98 ± 0.87 ^c^	211.43 ± 2.46 ^c^
GUP_HS_20		266.22 ± 0.25 ^b^	215.36 ± 1.16 ^c^

**Table 4:** Concentration of tentatively identified volatile compounds in the virgin olive oil samples and in those flavored with orange, orange by-product, and orange by-product with black pepper, expressed in mg/kg. Letters of significance refer to ANOVA, Tukey’s HSD (*p* ≤ 0.05). TEST_1, TEST_2 = olive oil control samples; AR = co-milled olive oil produced by milling olives and entire oranges; ST_AR = co-milled olive oil produced by milling olives and orange pomace; ST_AR_P = co-milled olive oil produced by milling olives with orange pomace and black pepper. Roman numbers indicate different tentatively identified stereoisomers forms. LRI = Linear Retention Index (Kovats Indexes).

Compound	TEST_1 mg/kg	TEST_2 mg/kg	AR mg/kg	ST_AR mg/kg	ST_AR_P mg/kg	LRI
3-Ethyl-1,5-octadiene I	0.08 ± 0.00 ^B^	0.29± 0.04 ^A^	n.d.	n.d.	n.d.	961
3-Ethyl-1,5-octadiene II	1.51 ± 0.07 ^A^	1.23 ± 0.19 ^A^	n.d.	n.d.	n.d.	968
3-Ethyl-1,5-octadiene III	1.32 ± 0.14 ^A^	1.26± 0.23 ^A^	n.d.	n.d.	n.d.	1012
α-Pinene	n.d.	n.d.	8.61 ± 0.59 ^B^	6.96 ± 0.64 ^B^	23.49 ± 0.79 ^A^	1022
3-Thujene	n.d.	n.d.	0.79 ± 0.09 ^B^	0.63 ± 0.07 ^B^	4.58 ± 0.26 ^A^	1028
1-Penten-3-one	1.58 ± 0.06 ^C^	0.90 ± 0.08 ^C^	1.52 ± 0.10 ^B^	2.05 ± 0.29 ^B^	3.36 ± 0.24 ^A^	1035
Hexanal	0.51 ± 0.02 ^C^	0.43 ± 0.04 ^C^	0.51 ± 0.06 ^B^	1.28 ± 0.06 ^A^	n.d.	1094
β-Pinene II	n.d.	n.d.	n.d.	n.d.	24.29 ± 1.27	1109
Sabinene	n.d.	n.d.	5.82 ± 0.60 ^B^	4.08 ± 0.34 ^B^	19.91 ± 2.14 ^A^	1125
3-Carene	n.d.	n.d.	0.66 ± 0.06 ^B^	1.04 ± 0.08 ^B^	43.48 ± 1.39 ^A^	1154
β-Myrcene	n.d.	n.d.	108.43 ± 6.42 ^B^	89.52 ± 7.57 ^C^	138.66 ± 2.63 ^A^	1171
4-Carene	n.d.	n.d.	0.70 ± 0.12 ^B^	n.d.	1.61 ± 0.12 ^A^	1186
Limonene	0.22 ± 0.01 ^D^	10.39 ± 1.24 ^C^	1336.32 ± 92.92 ^A^	1088.06 ± 83.82 ^B^	1475.72 ± 18.05 ^A^	1209
β-Phellandrene	n.d.	n.d.	6.45 ± 1.17 ^B^	5.12 ± 0.18 ^B^	11.04 ± 0.92 ^A^	1216
(*E*)-2-Hexenal	2.74 ± 0.4 ^C^	15.54 ± 1.35 ^B^	44.28 ± 6.99 ^A^	15.04 ± 2.40 ^B^	26.63 ± 0.29 ^B^	1237
γ-Terpinene	n.d.	n.d.	n.d.	n.d.	2.84 ± 0.13	1254
α-Terpinene	n.d.	n.d.	0.79 ± 0.10 ^A^	0.51 ± 0.04 ^B^	n.d.	1255
(Z)-β-Ocimene	0.59 ± 0.02 ^B^	0.52 ± 0.04 ^B^	n.d.	n.d.	2.83 ± 0.37 ^A^	1261
(E)-β-Ocimene	n.d.	n.d.	1.76 ± 0.11 ^A^	1.62 ± 0.09 ^A^	n.d.	1263
m-Cymene	n.d.	n.d.	n.d.	1.04 ± 0.18	n.d.	1282
Terpinolene	n.d.	n.d.	1.31 ± 0.04 ^B^	n.d.	3.34 ± 0.26 ^A^	1293
Octanal	n.d.	n.d.	6.35 ± 1.23 ^A^	4.33 ± 0.75 ^A^	1.61 ± 0.18 ^B^	1302
Geranyl nitrile	n.d.	n.d.	0.90 ± 0.10 ^A^	0.44 ± 0.04 ^B^	0.72 ± 0.13 ^A^	1315
(Z)-2-Penten-1-ol	0.36 ± 0.03 ^CD^	0.18 ± 0.01 ^D^	0.41 ± 0.02 ^C^	0.88 ± 0.10 ^B^	1.28 ± 0.11 ^A^	1333
1-Hexanol	0.05 ± 0.01 ^C^	0.10 ± 0.01 ^C^	0.35 ± 0.06 ^B^	1.29 ± 0.20 ^A^	1.28 ± 0.04 ^A^	1364
(*Z*)-3-Hexen-1-ol	n.d.	n.d.	n.d.	n.d.	3.03 ± 0.33	1393
(*E*)-3-Hexen-1-ol	0.49 ± 0.00 ^C^	0.05 ± 0.00^D^	n.d.	2.89 ± 0.07 ^A^	1.60 ± 0.11 ^B^	1398
(*E*)-2-Hexen-1-ol	0.05 ± 0.00 ^C^	0.56 ± 0.04 ^B^	n.d.	2.74 ± 0.16 ^A^	n.d.	1446
(*E*,*E*)-2,4-Hexadienal	0.13 ± 0.04 ^C^	0.10 ± 0.02 ^C^	0.59 ± 0.08 ^A^	0.25 ± 0.05 ^B^	n.d.	1460
Citronellal	n.d.	n.d.	n.d.	0.84 ± 0.02 ^B^	1.31 ± 0.02 ^A^	1489
Copaene	n.d.	n.d.	n.d.	n.d.	1.66 ± 0.12	1496
Decanal	n.d.	n.d.	n.d.	4.67 ± 0.81 ^A^	3.84 ± 0.72 ^A^	1511
Linalool	n.d.	n.d.	1.45 ± 0.00 ^B^	0.15 ± 0.05 ^B^	9.90 ± 0.77 ^A^	1556
Linalyl formate	n.d.	n.d.	n.d.	9.08 ± 1.34	n.d.	1587

**Table 5:** Concentration of tentatively identified volatile compounds in the virgin olive oil sample and the related flavored oils with hemp seeds at different ratios, expressed in mg/kg. Letters of significance refer to ANOVA, Tukey’s HSD (*p* ≤ 0.05). TEST_3 = olive oil control sample; HTEST_1 = cold-pressed hemp seed oil control sample; IUP_HS_10 = co-milled olive oil produced by milling olives with 10% intact unpeeled hemp seeds; IUP_HS_20 = co-milled olive oil produced by milling olives with 20% intact unpeeled hemp seeds; GUP_HS_10 = co-milled olive oil produced by milling olives with 10% ground unpeeled hemp seeds; GUP_HS_20 = co-milled olive oil produced by milling olives with 20% ground unpeeled hemp seeds. Roman numbers indicate different tentatively identified stereoisomer forms. LRI = Linear Retention Index (Kovats Indexes).

Compound	TEST_3 mg/kg	HTEST_1 mg/kg	IUP_HS_10 mg/kg	IUP_HS_20 mg/kg	GUP_HS_10 mg/kg	GUP_HS_20 mg/kg	LRI
2-Methyl-butanal	0.12 ± 0.00 ^c^	n.d.	0.12 ± 0.00 ^c^	0.11 ± 0.00 ^c^	0.16 ± 0.00 ^a^	0.14 ± 0.01 ^b^	912
3-Methyl-butanal	0.09 ± 0.00 ^c^	n.d.	0.10 ± 0.00 ^b,c^	0.11 ± 0.00 ^b^	0.11 ± 0.01 ^b,c^	0.13 ± 0.01 ^a^	917
3-Pentanone	0.08 ± 0.00 ^a^	n.d.	0.08 ± 0.02 ^a^	0.11 ± 0.02 ^a^	0.08 ± 0.01 ^a^	0.10 ± 0.01 ^a^	989
∝-Pinene	n.d.	3.49 ± 0.32 ^a^	0.25 ± 0.01 ^c,d^	0.62 ± 0.12 ^b,c^	0.34 ± 0.01 ^b,c,d^	0.70 ± 0.04 ^b^	1029
Hexanal	0.41 ± 0.01 ^c^	0.03 ± 0.00 ^d^	0.55 ± 0.01 ^b,c^	0.75 ± 0.014 ^a^	0.73 ± 0.02 ^a^	0.70 ± 0.03 ^a,b^	1100
β-Pinene I	n.d.	1.20 ± 0.12 ^a^	0.18 ± 0.02 ^c,d^	0.44 ± 0.08 ^b^	0.12 ± 0.00 ^c,d^	0.24 ± 0.02 ^b,c^	1133
3-Carene	n.d.	0.24 ± 0.03 ^a^	n.d.	n.d.	n.d.	0.06 ± 0.01 ^b^	1162
β-Pinene II	n.d.	2.21 ± 0.22 ^a^	0.18 ± 0.01 ^c,d^	0.42 ± 0.07 ^b,c^	0.23 ± 0.01 ^b,c^	0.49 ± 0.03 ^b^	1190
Limonene	n.d.	0.67 ± 0.07 ^a^	0.06 ± 0.00 ^c,d^	0.13 ± 0.02 ^c^	0.08 ± 0.01 ^c,d^	0.16 ± 0.00 ^b^	1209
3-Methyl-butanol	0.29 ± 0.01 ^a^	n.d.	0.31 ± 0.00 ^a^	0.26 ± 0.04 ^a^	0.20 ± 0.01 ^b^	0.29 ± 0.01 ^a^	1222
(*E*)-2-Hexenal	0.13 ± 0.00 ^c^	n.d.	0.14 ± 0.01 ^c^	0.15 ± 0.03 ^c^	0.42 ± 0.03 ^a^	0.22 ± 0.02 ^b^	1240
(*Z*)-β-Ocimene	0.25 ± 0.01 ^b^	0.35 ± 0.03 ^a^	0.26 ± 0.01 ^b^	0.20 ± 0.03 ^b^	0.19 ± 0.02 ^b^	0.22 ± 0.02 ^a^	1264
1-Hexanol	0.51 ± 0.01 ^b^	0.52 ± 0.05 ^b^	0.59 ± 0.02 ^b^	0.52 ± 0.09 ^b^	0.72 ± 0.03 ^a^	0.55 ± 0.03 ^b^	1362
(*Z*)-3-Hexenol	1.03 ± 0.01 ^b^	n.d.	0.99 ± 0.24 ^b,c^	0.54 ± 0.10 ^d^	1.51 ± 0.07 ^a^	0.70 ± 0.03 ^c,d^	1402
Caryophyllene	n.d.	0.32 ± 0.06 ^a^	0.03 ± 0.00 ^b,c^	0.07 ± 0.02 ^b,c^	0.05 ± 0.02 ^b,c^	0.10 ± 0.02 ^b^	1616

**Table 6:** Median values of the intensity of the sensory attributes perceived by the panel on olive oils flavored with orange, orange by-product, and orange by-product and black pepper, as well as their control samples. TEST_1, TEST_2 = olive oil control samples; AR = co-milled olive oil produced by milling olives and entire oranges; ST_AR = co-milled olive oil produced by milling olives and orange pomace; ST_AR_P = co-milled olive oil produced by milling olives with orange pomace and black pepper.

	Attribute (Median Intensity)						
Sample	Fruity	Bitter	Pungent	Citrus	Pepper	Red Fruits	Peas
TEST_1	3.0	2.5	2.5				2.4
TEST_2	3.0	3.8	4.7			3.1	
AR	1.8	3	3	3.6			
ST_AR	2	3.1	2.5	3.6			
ST_AR+P	1.8	3.	3.1	2.7	3.9		

## Data Availability

The original data presented in the study are openly available in Zenodo at the following doi: https://doi.org/10.5281/zenodo.14655315.
